# The anillin-related Int1 protein and the Sep7 septin collaborate to maintain cellular ploidy in *Candida albicans*

**DOI:** 10.1038/s41598-018-20249-9

**Published:** 2018-02-02

**Authors:** Sara Orellana-Muñoz, Encarnación Dueñas-Santero, Yolanda Arnáiz-Pita, Francisco del Rey, Jaime Correa-Bordes, Carlos R. Vázquez de Aldana

**Affiliations:** 1 Instituto de Biología Funcional y Genómica, IBFG-CSIC. Universidad de Salamanca, Salamanca, Spain; 2 Departamento de Ciencias Biomédicas, Universidad de Extremadura, Badajoz, Spain

## Abstract

Variation in cell ploidy is a common feature of *Candida albicans* clinical isolates that are resistant to the antifungal drug fluconazole. Here, we report that the anillin-related protein Int1 interacts with septins for coupling cytokinesis with nuclear segregation. Loss of Int1 results in a rapid disassembly of duplicated septin rings from the bud neck at the onset of actomyosin ring contraction. Strikingly, this has no major impact on cytokinesis and septum formation. However, Int1 genetically interacts with the Sep7 septin, maintaining the diffusion barrier at the bud neck and guarantying a faithful nuclear segregation. Indeed, *int1*ΔΔ *sep7*ΔΔ mutant cells, in contrast to *int1*ΔΔ *cdc10*ΔΔ, undergo a premature activation of mitotic exit prior to the alignment of the mitotic spindle with the division axis, producing large multinucleated cells. Some of these multinucleated cells arise from trimeras similar to those observed upon fluconazole exposure. Finally, the defects in nuclear segregation could be in part due to the inability to maintain the Lte1 mitotic exit activator at the cortex of the daughter cell. These results suggest that Int1 and Sep7 play a role in maintaining genome stability by acting as a diffusion barrier for Lte1.

## Introduction

At the beginning of a new cell cycle, the cells must coordinate a series of events that result in DNA replication, duplication of the spindle polar bodies (SPBs, equivalent to centrosome in animal cells) and formation of the daughter cell. The newly duplicated genetic material must be segregated in a reliably and closely regulated manner. Errors in this process result in the exclusion of chromosomes from the daughter cell and an increase in the genetic material in the mother. Polyploidy can lead to an increase in the demand for DNA replication and chromosome maintenance machineries, which can have severe consequences for the cell^1^. The correct separation of the genomes during mitosis requires a precise coordination between mitotic spindle orientation towards the cortex and cytokinesis. The septins play important roles in these processes by marking the position of the cortex where the bud is located, while anillin (a conserved animal protein) is involved in cytokinesis^2,3^.

*S. cerevisiae* contains five mitotic septins, Cdc3, Cdc12, Cdc10, Cdc11 and Shs1 (also known as Sep7). Septins form heteropolymers that associate with each other to form filaments^4^. Prior to bud emergence, the septins form patches in the cortex that are transformed into a ring^5,6^. From bud emergence to the end of mitosis, septin filaments are organized as an hourglass structure at the bud neck, helping to maintain cell polarity by acting as a barrier between the mother and daughter cells. This structure also serves as a scaffold for other cytoskeletal components such as the actomyosin ring (AMR)^7,8^. At the onset of cytokinesis, the septins rings duplicate and delimit a specialized compartment^9^. Cytokinesis must be coordinated with other mitotic processes to ensure that cell separation occurs only after the chromosomes have been segregated. The septins are necessary for the correct positioning of the spindle relative to the division plane. Septins regulate diverse processes, such as spindle positioning or the mitotic checkpoint^10,11^, and coordinate spindle positioning with cell division providing spatial cues that guide spindle movements^12,13^. When the spindle is incorrectly aligned, the Spindle POsitioning Checkpoint (SPOC) stops mitosis and provides time for its reorientation, preventing the formation of anucleated and/or polyploid cells^14^. It has been shown that the *shs1*Δ and *cdc10*Δ mutants are defective in the SPOC when movement of the spindle into the daughter cell was delayed, leaving both nuclei in the mother cell^13^.

Anillin was first identified as an F-actin binding protein in *Drosophila melanogaster*^15^. It also binds to septins and type II myosin independently of F-actin, playing a role in the assembly and organization of the AMR^16^. *S. cerevisiae* contains three proteins that share homology with anillin. Boi1 and Boi2 are part of the NoCut pathway that coordinates cytokinesis with the completion of chromosome segregation^17^. The third homolog, Bud4, was identified through its role as a landmark protein that defines the future site of haploid cell division^18^. *bud4*Δ mutants lack double septin rings, although AMR assembly, cytokinesis and septum formation occur efficiently, suggesting that the double septin ring is dispensable for cytokinesis in *S. cerevisiae*^19–21^. Bud4 plays a role in septin ring organization during bud growth, since the *bud4*Δ *shs1*Δ mutant has elongated buds and is defective in septin organization at 18 °C^20,22^.

*Candida albicans* is the most commonly isolated human pathogen able to withstand genetic and epigenetic changes, often in response to specific environmental factors. It has been previously reported that *C. albicans* has two anillin-related homologues in the genome. There is only one homologue to *S. cerevisiae* Boi1 and Boi2, known as Boi2, which is required for vacuolar fusion^23^. The second is known as Int1, which is homologue to Bud4 and contains the same PH (Pleckstrin Homology) and anillin (also named DUF1709) domains^21,24,25^. In *S. cerevisiae*, the different Bud4 domains have distinct functions. The PH domain interacts with negatively charged membrane phosphoinositides, the C-terminus (PH and DUF1709) is involved in the organization of septins, whereas Bud4 function after septin ring duplication requires both the N- and C-termini. This suggests that several regions of the protein cooperate to promote a strong interaction with septins, which helps to prevent the loss of subunits during the transition to double septin rings^22^. There are few data concerning the function of Int1 in *C. albicans*. Int1 co-localizes with septins throughout the cell cycle and may play a role in bud site selection^26^. Int1 is also important for hyphal morphogenesis, adhesion and virulence. *int1*ΔΔ mutants have a reduced ability to form hyphae in Milk-Tween and Spider medium, but form apparently normal hyphae in the presence of serum^25^. In addition, *int1*ΔΔ mutants have attenuated virulence in systemic candidiasis mouse models. When *INT1* is expressed in *S. cerevisiae*, it generates cells that grow with a filamentous morphology that contain abnormal spirals in which Int1 and septins co-localize and interact^27^.

In this report, the function of Int1 in *C. albicans* is characterized. Similar to what has been described in *S. cerevisiae*, septin rings are unstable after duplication in *int1*ΔΔ cells, although the cells are able to complete cytokinesis and septum assembly in the absence of duplicated septin rings. Interestingly, it was found that the regulatory septin Sep7 and Int1 perform an essential function in the maintenance of the cellular ploidy, since *int1*ΔΔ *sep7*ΔΔ mutants have defects in nuclear segregation that result in the formation of polynucleated cells. This defect could be in part due to the inability to maintain the activator of mitotic exit Lte1 at the cortex of the daughter cell. This suggests that the combined action of Int1 and Sep7 is critical for maintaining the integrity of the septin ring and for avoiding abnormal nuclear segregation.

## Results

### Int1 localizes with septins throughout the cell cycle

**Figure 1:**
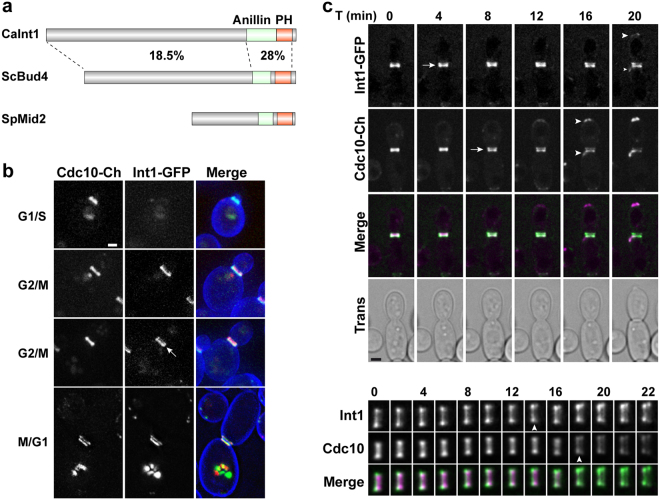
Int1 and Cdc10 colocalize along the cell cycle. (**a**) Schematic representation of the *C. albicans* Int1, *S. cerevisiae* Bud4 and *S. pombe* Mid2 proteins. The position of the conserved anillin (green) and PH (red) domains is indicated. The percentage of identity (%) between Int1 and Bud4 in the different regions is indicated. (**b**) Localization of Int1-GFP and Cdc10-mCherry during yeast growth. Exponential growing cultures of the *INT1-GFP CDC10-mCherry* strain (OL2262) were stained with calcofluor. The images are the maximum projection of 10 planes and show the Int1-GFP and Cdc10-mCherry channels and the merged image (Int1-GFP, green; Cdc10-mCherry, red; calcofluor, blue). Scale bar, 2 μm. (**c**) Time-lapse analysis of Int1-GFP and Cdc10-mCherry during yeast growth. The images are the maximum projection of 3 planes and show Int1-GFP (green), Cdc10-mCherry (magenta) and the merged channel. Images were acquired at the indicated time points (minutes). Arrows indicate duplication of the rings, while arrowheads mark the assembly of the new budding site. Scale bar: 2 μm. Below the image, a magnification of the septum region is shown.

Int1 is an orthologue of *S. cerevisiae* Bud4, sharing a low level of overall identity (22%), although it is higher in the region containing the two conserved domains (around 28%)(Fig. 1a). To analyse Int1 localization along the cell cycle in *C. albicans*, *INT1* was tagged with the green fluorescent protein (GFP) at the 3′ end in cells carrying *CDC10-mCherry*. Microscopic analysis of the cells showed that Int1-GFP co-localized with septins at all stages of the cell cycle, first appearing at the budding site as a single ring that duplicated at the onset of cytokinesis (Fig. 1b). Interestingly, there were temporal differences in the assembly of Int1 and septins at the cell cortex. Thus, assembly of the septin ring at the new budding site occurred before Int1 incorporation, since cells with septin caps and without Int1 signal were often observed, while the opposite was never found. In contrast, at the onset of cytokinesis, duplication of the Int1 ring preceded septin ring splitting, since cells with duplicated Int1 rings and single septin ring were observed. During hyphal growth, Int1 also co-localized with septins at the site of septum formation with the same temporal order (Supplementary Figure S1). Therefore, these observations show that the temporal regulation of Int1 and septin dynamics at the division plane is not modified by hyphal-inducing cues.

To analyse the dynamics of ring duplication with more detail, time-lapse microscopy was used. The results confirmed that Int1 ring duplication occurred 2–4 minutes before septin ring duplication (Fig. 1c, arrows). However, Cdc10-mCherry assembled at the bud site before Int1-GFP upon bud emergence (Fig. 1c, arrowheads). We also tested whether Int1 localization was dependent on septins. Int1-GFP localization was analysed in *cdc10*ΔΔ and *sep7*ΔΔ mutants. Int1-GFP localized to the bud neck in both mutants, similarly to the wild-type strain (Supplementary Figure S1). However, quantification of Int1-GFP intensity indicated a reduction in the signal compared to the wild type, indicating a partial dependence of septins for Int1 bud neck localization (Supplementary Figure S1).

### Int1 is necessary to stabilize the septin ring after duplication during cytokinesis

**Figure 2:**
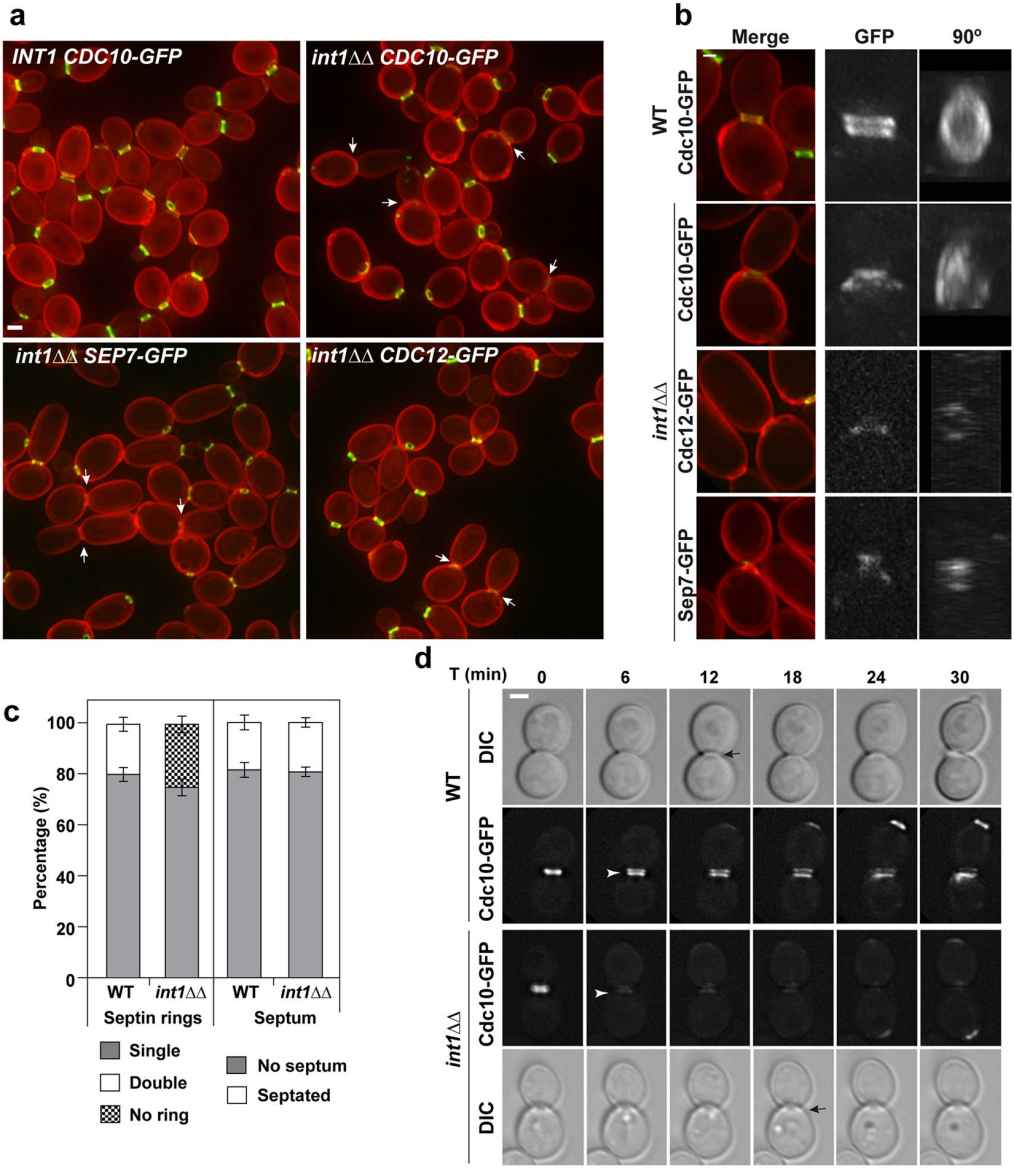
The *int1*ΔΔ mutant has no double septin rings but completes cytokinesis correctly. (**a**) Images of *CDC10-GFP* (OL2243), *int1*ΔΔ *CDC10-GFP* (OL2316), *int1*ΔΔ *CDC12-GFP* (OL2732) and *int1*ΔΔ *SEP7-GFP* (OL2734) cells during yeast growth. The images are the maximum projection of 10 planes acquired every 0.4 μm and show the merged signal of septin-GFP (green) and calcofluor staining (red). Scale bar, 2 μm. Arrows indicate *int1*ΔΔ cells with visible septum (by calcofluor staining). (**b**) Detail of duplicated septin rings in wild-type and *int1*ΔΔ yeast cells. The images show the Cdc10-GFP fluorescence (green) and calcofluor staining (red). The histogram for *int1*ΔΔ cells has been adjusted to facilitate visualization of septin structures. (**c**) *int1*ΔΔ mutants have no duplicated septin rings, although the percentage of septated cells is similar to the wild type. The left graph shows the percentage of cells with single septin ring (grey), double septin ring (white) or without septins (black) in the wild-type and the *int1*ΔΔ mutant. The right graph shows the percentage of septated cells (white) or cells without septum (grey) in the same strains. The graph represents the average of four independent experiments ± s.e.m., and at least 200 cells were counted in each experiment. (**d**) The septin ring is unstable after duplication in *int1*ΔΔ cells. Time lapse analysis of Cdc10-GFP in wild-type and *int1*ΔΔ cells during cytokinesis. Images of differential interference contrast (DIC) and Cdc10-GFP were acquired at the indicated time points (minutes). The GFP image is the maximum projection of 10 z-planes. White arrowheads indicate the moment of septin ring duplication whereas black arrows in DIC images indicate the septum. Scale bar, 2 μm.

The phenotype of cells lacking Int1 was characterized. As previously described^25^, loss of *INT1* caused no detectable growth or morphological defect in yeasts and the mutant was able to form hyphae in response to serum. Thus, we investigated whether Int1 was required for septin ring stability. Cdc10-GFP rings were analysed in cells lacking Int1. In contrast to wild-type cells, no double Cdc10-GFP rings were observed in *int1*ΔΔ cells (Fig. 2a, arrows). However, remnants of Cdc10-GFP were detected at both sides of the neck when the histogram was adjusted (Fig. 2b). Similar results were obtained for Cdc12-GFP or Sep7-GFP (Fig. 2a,b), indicating that the absence of Int1 affects the stability of duplicated septin rings.

The percentage of cells with single or double septin rings was determined. Although around 20% of wild-type cells had duplicated septin rings, no duplicated rings were observed in *int1*ΔΔ cells (Fig. 2c, left graph). Strikingly, the percentage of cells with septa (by calcofluor staining) was similar in both strains (Fig. 2c, right graph). Similar results were observed during hyphal development, since no subapical septin rings were found in *int1*ΔΔ hyphae (Supplementary Figure S2). Therefore, these observations suggest that Int1 plays a specific role in the stabilization of septin rings after duplication in both types of growth in *C. albicans*, although this process is dispensable for the assembly of the septum during cytokinesis.

The kinetics of septin ring duplication in wild-type and *int1*ΔΔ cells was analysed by time-lapse microscopy. In wild-type cells, duplication of the septin ring slightly preceded the assembly of the septum (Fig. 2d, upper panels), and remained stable for more than 12 min before its disassembly in G1. However, loss of Int1 caused the disassembly of the septin ring in large-budded cells at the time of septin ring duplication (Fig. 2d, lower panels).

### Actomyosin ring contraction is slightly delayed by the absence of Int1

**Figure 3:**
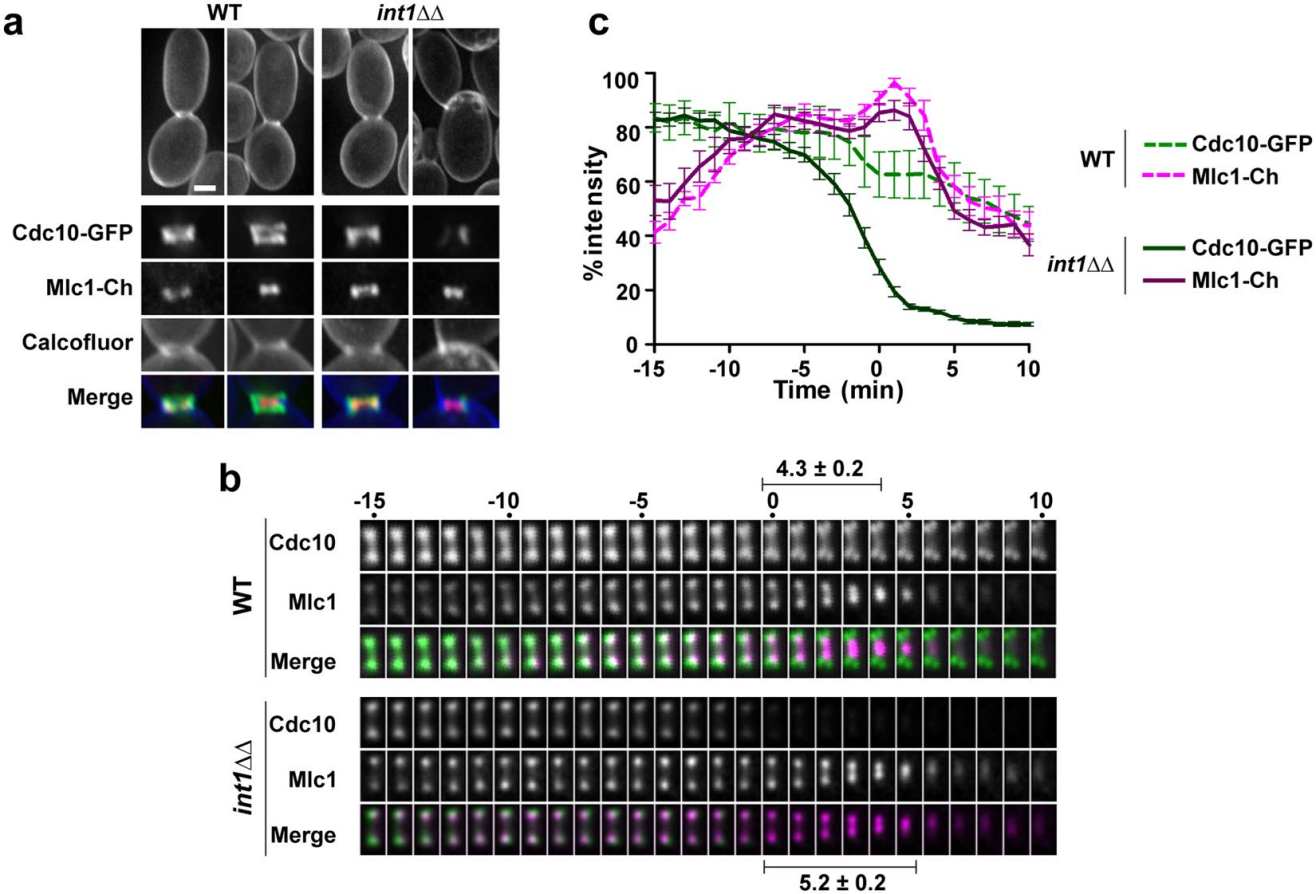
Actomyosin ring constriction can occur in the absence of a duplicated septin ring. (**a**) Localization of Cdc10-GFP and Mlc1-mCherry in wild type (OL2338) and in the *int1*ΔΔ mutant (OL2328) cells. The image shows examples of cells before and during AMR constriction. Below the cells, a magnification of the neck region is shown. The merged image contains the Cdc10-GFP (green), Mlc1-mCherry (red) and calcofluor (blue) signals. Scale bar, 2 μm. (**b**) Dynamics of AMR contraction. Left: Time-lapse images of Cdc10-GFP and Mlc1-mCherry. The images are the maximum projection of 10 z-planes acquired at the indicated times. The merged image shows Cdc10-GFP (green) and Mlc1-mCherry (magenta). (**c**) Quantification of the average intensity of Cdc10-GFP (green) and Mlc1-mCherry (magenta) in the wild type (dashed line) and *int1*ΔΔ mutant (solid line). Time zero indicates the beginning of AMR contraction. The graph represents the average intensity of the different channels (Cdc10-GFP or Mlc1-mCherry) of 20 independent cells from the wild type or the *int1*ΔΔ mutant ± s.e.m.

AMR contraction was studied by tagging the myosin light chain (Mlc1) with mCherry in strains carrying *CDC10-GFP*. Microscopic inspection of wild-type cells showed that the AMR assembled on the septin collar and contracted after septin ring duplication (Fig. 3a). In the *int1*ΔΔ mutant, the structure of the AMR before septin duplication was similar to that of the wild type, and AMR contracted in the absence of double septin rings (Fig. 3a). Therefore, the septin collar is functional to assemble the AMR in *int1*ΔΔ cells, which is able to contract in the absence of a double septin ring.

The kinetics of AMR contraction was analysed by time-lapse microscopy in these strains. The average intensity of Cdc10-GFP and Mlc1-mCherry signals at the bud neck was quantified over time in both strains (n = 20), as shown in Fig. 3c, where time 0 indicates the onset of AMR contraction. The data shows that loss of Int1 did not affect the intensity of Mlc1 at the bud neck. However, the stability of Cdc10 was clearly modified, since the GFP signal started to decrease several minutes before the onset of AMR contraction, dropping approximately to 20% of the initial intensity at time 0. When the AMR contraction was analysed, it was found to be slightly delayed due to the loss of Int1. Thus, the average contraction time was 4.3 ± 0.2 min for the wild-type strain, but 5.2 ± 0.2 min in *int1*ΔΔ cells, a difference that was statistically significant (n = 20; p = 0.0068; Fig. 3b).These results indicate that, despite the absence of duplicated septin rings, *int1*ΔΔ cells are capable of contracting the AMR, although with a slightly delayed kinetics compared to the wild-type strain.

### The N- and C-terminal regions of Int1 have different functions

**Figure 4:**
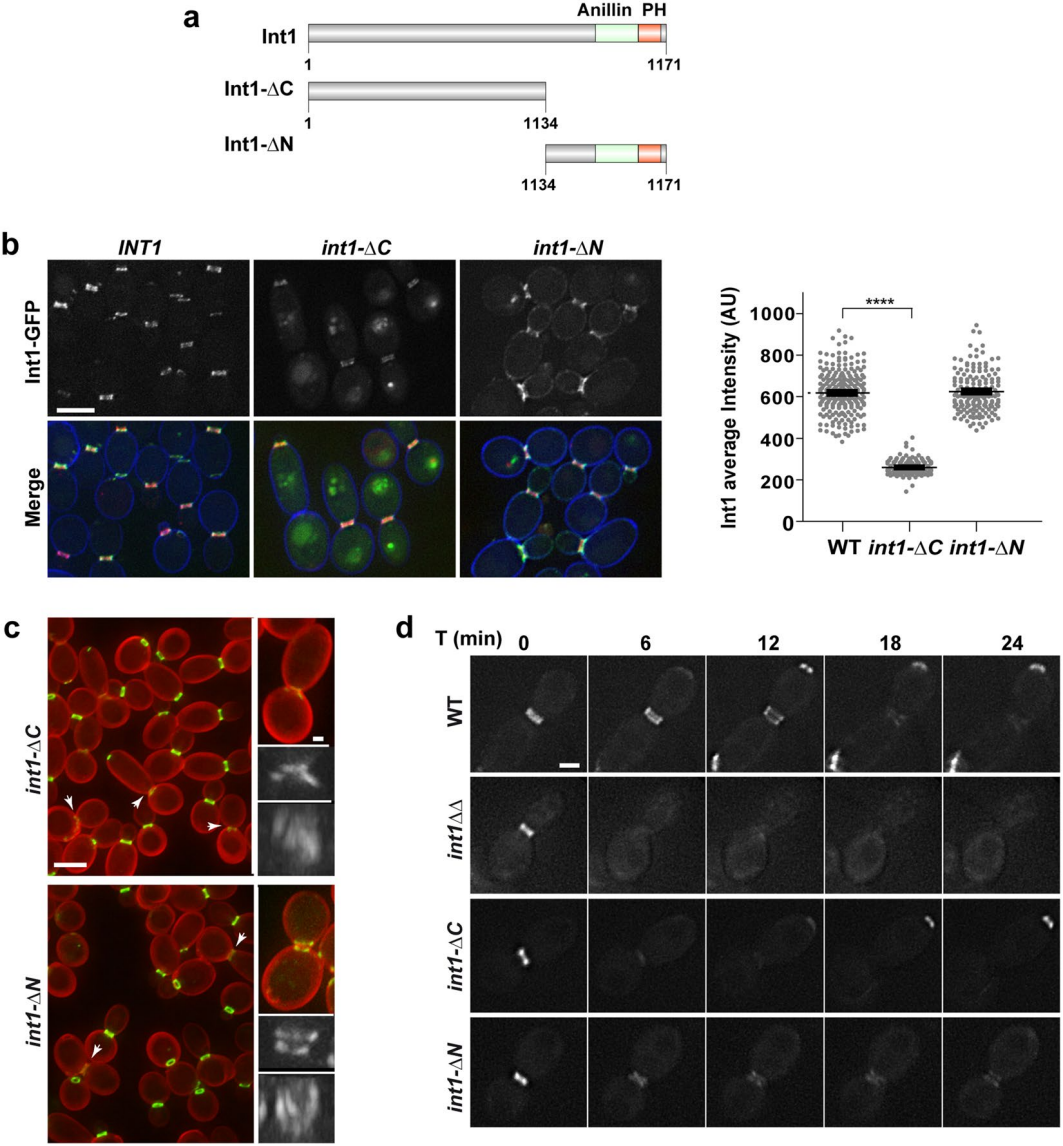
The N- and C-terminal regions of Int1 have different contributions to its function. (**a**) Schematic representation of Int1, Int1-ΔC and Int1-ΔN proteins. The conserved anillin (green) and PH (red) regions are indicated. (**b**) Fluorescence images of Int1-GFP (green) and the merged channel (with Int1-GFP in green, Cdc10-mCherry in red and calcofluor in blue) of *INT1-GFP CDC10-mCherry* (OL2262), *int1-*Δ*C-GFP/int1*Δ *CDC10-mCherry* (OL2278) and *int1-*Δ*N-GFP/int1*Δ *CDC10-mCherry* (OL2280) cells. The images are the maximum projection of 3 z-planes. The histogram of Int1-GFP images has the same scale, whereas in the merged image the green channel has been adjusted to facilitate visualization. Scale bar: 2 μm. The graph represents the average intensity of three independent experiments ± s.e.m (n > 50 Int1-GFP rings in each strain; ****p-value < 0.0001). (**c**) Phenotype of the *int1-*Δ*C CDC10-GFP* (OL2193) and *int1-*Δ*N CDC10-GFP* (OL2304) strains during yeast growth. Maximum projection of 10 planes of Cdc10-GFP (green) and calcofluor (red) are shown. Arrows indicate cells with visible septum (according to calcofluor). Scale bar, 5 μm. The details show *int1-*Δ*C* and *int1-*Δ*N* cells with duplicated rings. The images show the Cdc10-GFP fluorescence (green) and calcofluor staining (red). The maximum projection of Cdc10-GFP and the rotated image are shown. Scale bar, 2 μm. **(d)** Time-lapse analysis of septin ring duplication in *int1-*Δ*C CDC10-GFP* (OL2193) and *int1-*Δ*N CDC10-GFP* (OL2304) yeast cells. The images are the maximum projection of 10 z-planes at the indicated times (minutes). Scale bar, 2 μm. The wild-type *CDC10-GFP* (OL2243) and *int1*ΔΔ *CDC10-GFP* (OL2316) strains have been included to facilitate the comparison of the relative intensities.

To identify functional domains in Int1, two truncated alleles were constructed: one where the C-terminal region containing the anillin and PH domains was deleted (Int1-ΔC, aa 1-1134) and another which lacked the N-terminal region (Int1-ΔN, aa 1134-1711) (Fig. 4a). These truncated alleles were transformed into the *INT1/int1*Δ strain, giving rise to strains *int1-*Δ*C/int1*Δ and *int1-*Δ*N/int1*Δ, which expressed the truncated forms under its native promoter as the sole source of Int1 protein.

The localization of Int1-ΔN and Int1-ΔC was examined by tagging them with GFP in *INT1*/*int1*Δ cells containing Cdc10-mCherry. Both truncated Int1 proteins co-localized with Cdc10-mCherry at the bud neck (Fig. 4b). However, quantification of the Int1-GFP signal showed that loss of the C-terminal domain (Int1-ΔC) produced a 3-fold reduction of the protein at the bud neck, whereas the signal of the Int1-ΔN protein was similar to the wild-type (Fig. 4b, graph). This result indicates that the C-terminal domain of Int1 is required to stabilize the protein at the bud neck.

The stability of the septin ring in *int1-*Δ*N* and *int1-*Δ*C* strains was studied. Deletion of the C-terminal domain caused the absence of duplicated septin rings, similar to the phenotype of the *int1*ΔΔ mutant (Fig. 4c, arrows), and only remnants of Cdc10-GFP could be observed. In contrast, cells expressing Int1-ΔN displayed duplicated septin rings normally assembled in large-budded cells, although approximately 20% of them were incomplete or poorly assembled (data not shown). Time-lapse experiments confirmed that septins disassembled from the bud neck at the time of duplication in the absence of the C-terminal domain of Int1. By contrast, the septin rings persisted after duplication in *int1-*Δ*N* cells, although the intensity was lower than in the wild type (Fig. 4d). Thus, these results indicate that the C-terminus of Int1 is essential to maintain the stability of double septin rings during cytokinesis.

### Int1 and Sep7 collaborate in the maintenance of the cellular ploidy

**Figure 5:**
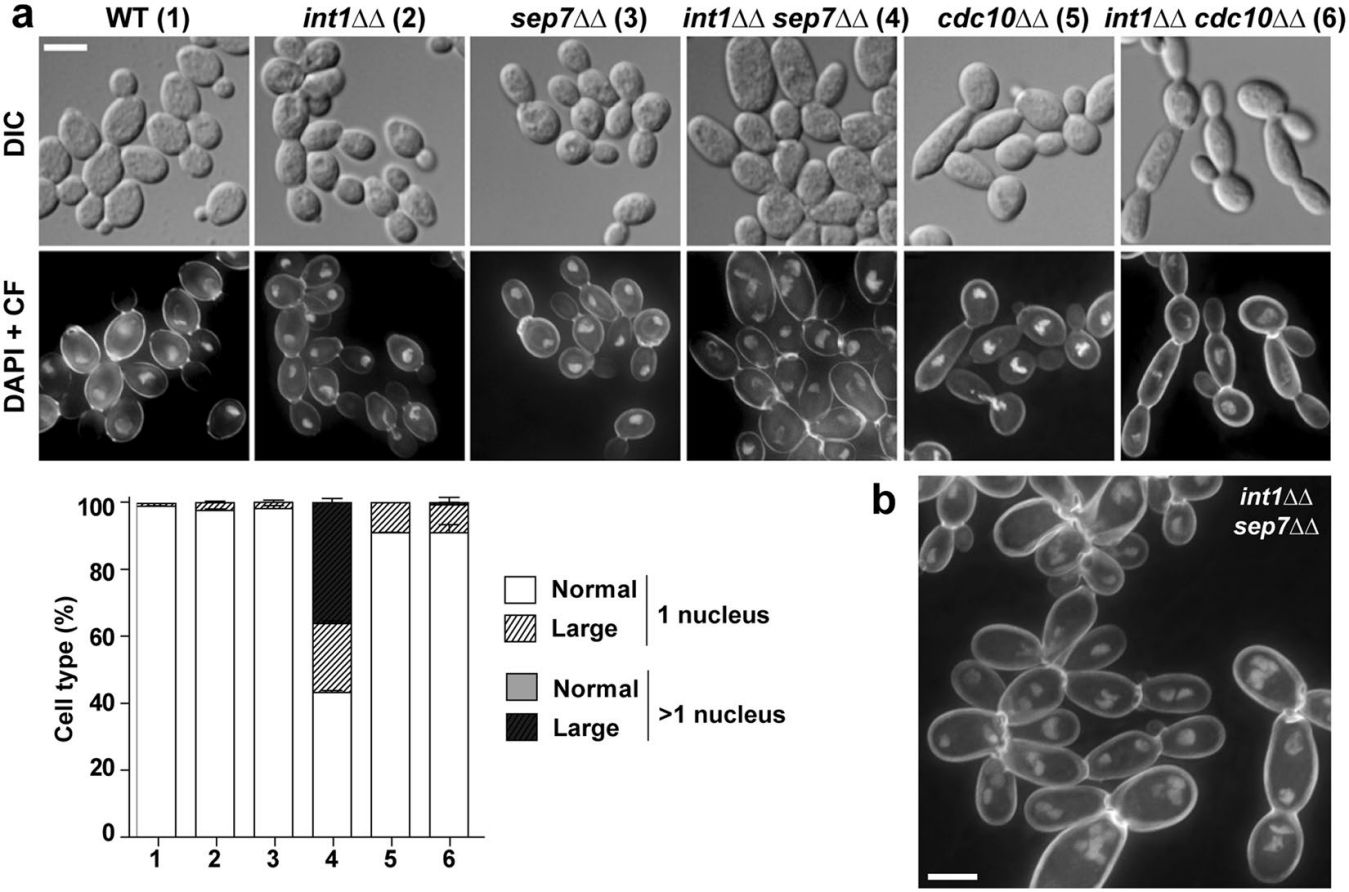
*int1*ΔΔ *sep7*ΔΔ mutants have increased number of nuclei. (**a**) Exponentially growing cells of the wild-type strain (WT, 1) and the *int1*ΔΔ (OL2314, 2), *sep7*ΔΔ (OL2138, 3), *int1*ΔΔ *sep7*ΔΔ (OL2509, 4), *cdc10*ΔΔ (OL2213, 5), *int1*ΔΔ *cdc10*ΔΔ (OL2532, 6) mutants were stained with DAPI and calcofluor (CF). Interferential difference contrast images (DIC) and the maximum projection of 3 z-planes showing DAPI and calcofluor staining are shown. Scale bar, 5 μm. The graph represents the average percentage of cells with one nucleus or more than one nucleus in the same strains (numbers) from three independent experiments with the standard error (s.e.m.), in which at least 150 cells were counted in each strain. Cells were classified in four categories: normal size with a single nucleus (white), normal size with more than 1 nucleus (grey), large size with a single nucleus (striped white) or large size with more than 1 nucleus (striped grey). Large cells were those with a volume 1.5 times higher than the average volume of a wild type strain (44 fL, see Supplementary Figure 3). The number of nuclear masses was determined by DAPI staining. (**b**) Detail of *int1*ΔΔ *sep7*ΔΔ cells stained with DAPI and calcofluor (CF). Scale bar, 5 μm.

To exacerbate the defects in septin organization, we investigated the genetic interactions between *int1*ΔΔ and the deletion of the non-essential septin gene *SEP7*. Sep7 plays a regulatory role for septin function in *S. cerevisiae* and *C. albicans*^28,29^. In solid media, growth of the *int1*ΔΔ *sep7*ΔΔ double mutant was similar to that of the single mutants at all temperatures tested (Supplementary Figure S3), suggesting no additive defects in the double mutant. Septin organization in *int1*ΔΔ *sep7*ΔΔ cells was analysed. The comparison of single and double septin rings showed no additive defects. The single rings of *int1*ΔΔ *sep7*ΔΔ cells had no apparent morphological defects, whereas double septin rings disassembled after duplication similar to *int1*ΔΔ cells (Supplementary Figure S3). However, microscopic examination of *int1*ΔΔ *sep7*ΔΔ cultures revealed the presence of abnormally large cells (around 58%) that were absent in the single mutants (Supplementary Figure S3). To determine whether this heterogeneity in size was associated with a defect in nuclear division, nuclear staining with 4,6-diamidino-2-phenylindole (DAPI) was performed. This showed that around 63% of the large *int1*ΔΔ *sep7*ΔΔ cells contained more than one nucleus (Fig. 5a,b).

Since septins function together as a complex, it could be expected that loss of other septins in cells lacking Int1 would also cause polyploidy. Thus, an *int1*ΔΔ *cdc10*ΔΔ double mutant was constructed. Interestingly, even though deletion of *CDC10* produced elongated buds as described^30^, and this defect was more pronounced in the *int1*ΔΔ *cdc10*ΔΔ double mutant, no multinucleated cells were observed in the latter mutant (Fig. 5a), suggesting that Sep7 can function independently of other septin subunits in the control of DNA content. Thus, Int1 and Sep7 prevent nuclear segregation defects, possibly acting by independent mechanisms.

The DNA content of log-phase yeast cells was determined using flow cytometry. Wild-type cells showed two populations corresponding to the G1-phase (2C DNA content) and G2-phase (4C DNA content) of the cell cycle, respectively. Flow cytometry data (Supplementary Figure S3) indicated that *cdc10*ΔΔ, *int1*ΔΔ, *sep7*ΔΔ and *int1*ΔΔ *cdc10*ΔΔ mutants showed a distribution of DNA content similar to that of wild-type cells. In contrast, the *int1*ΔΔ *sep7*ΔΔ mutant had a higher population of cells with DNA content > 8C. However, the possibility that a fraction of the cells with high DNA content are aneuploids from previous cell cycles cannot be ruled out. Therefore, these results indicate that cells with various nuclear masses observed by DAPI staining actually correspond to an increase in ploidy.

### The *int1*ΔΔ *sep7*ΔΔ mutant has defects in nuclear segregation

**Figure 6:**
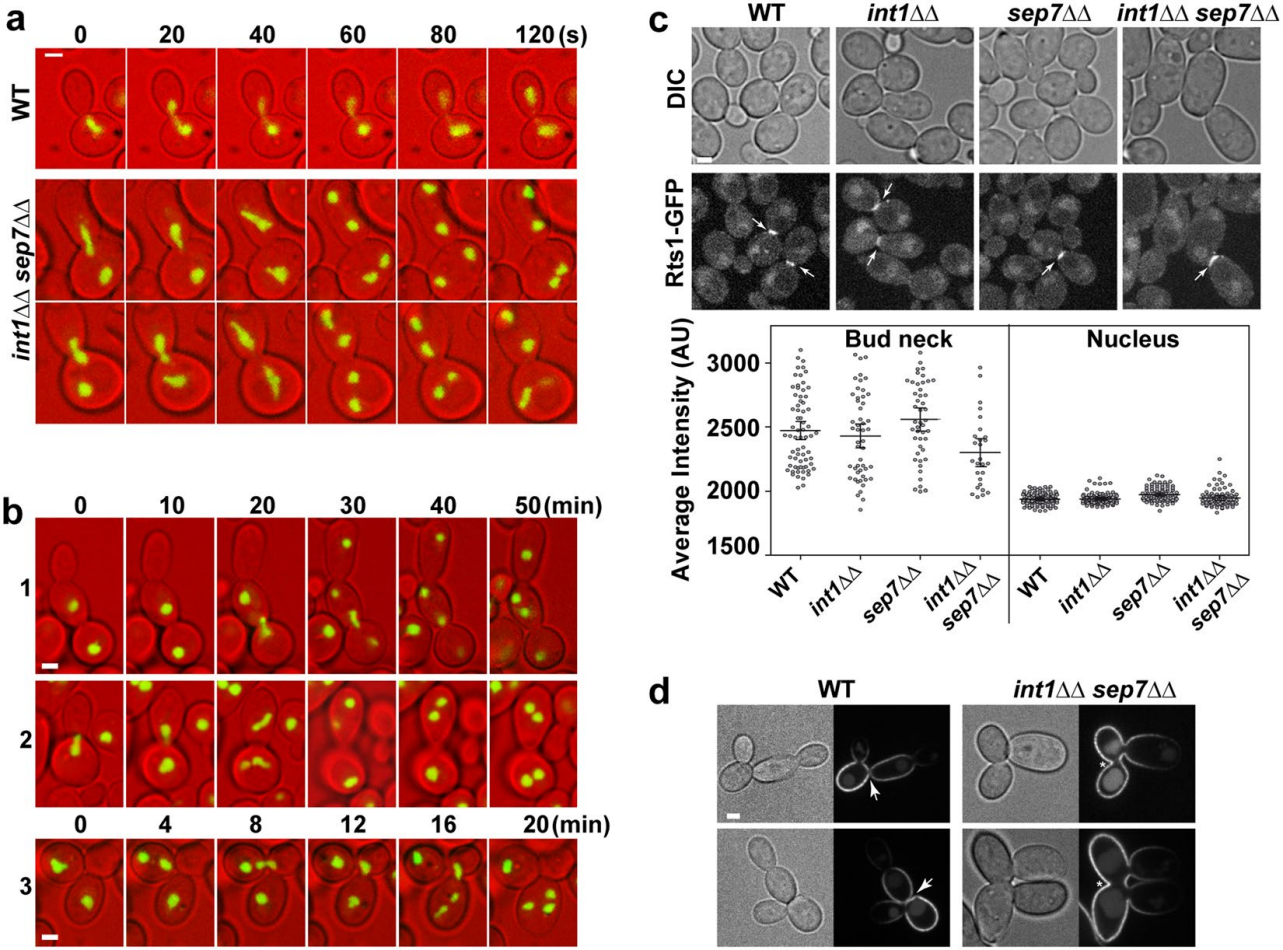
*int1*ΔΔ *sep7*ΔΔ mutants have abnormal nucleolar segregation. (**a**) Time-lapse analysis of Nop1 in the *NOP1-mCherry* (WT, OL2347) and *int1*ΔΔ *sep7*ΔΔ *NOP1-mCherry* (OL2514) strains. The images are the maximum projection of 3 z-planes and show DIC (red) and Nop1 fluorescence (green). Scale bar: 2 μm. (**b**) Additional examples of nucleolar segregation in the *int1*ΔΔ *sep7*ΔΔ *NOP1-mCherry* strain at the indicated time points (minutes). (**c**) Rts1 localization in *RTS1-GFP* (WT, OL2550), *int1*ΔΔ *RTS1-GFP* (OL2553), *sep7*ΔΔ *RTS1-GFP* (OL2552) and *int1*ΔΔ *sep7*ΔΔ *RTS1-GFP* (OL2555) strains. Images of differential interference contrast (DIC) and Rts1-GFP fluorescence. Images are the maximum projection of 3 z-planes. Arrows indicate septum localization of Rts1-GFP. Scale bar: 2 μm. The graph shows the average intensity ± s.e.m. of Rts1-GFP fluorescence from two independent transformants, in which at least twenty bud necks and nuclei were counted in each strain. (**d**) Images of Pma1-mCherry and DIC of *PMA1-mCherry* (OL2755) and *int1*ΔΔ *sep7*ΔΔ *PMA1-mCherry* (OL2783) strains. The images are a single focal plane at the centre of the cell. Arrows point to bud necks in which the double plasma membrane can be visualized. Asterisks indicate bud necks in which cytokinesis had not occurred. Scale bar: 2 μm.

For the correct segregation of genetic material, the cell must coordinate DNA replication, duplication of the spindle pole body (SPB) and bud emergence. Once duplicated, the genetic material must be segregated between the mother and daughter cells in an accurate manner to avoid the generation of aneuploid cells^1^. To elucidate the mechanisms contributing to the formation of polyploid cells in the *int1*ΔΔ *sep7*ΔΔ mutant, time-lapse microscopy was used to monitor the nucleolar marker Nop1. In wild-type large-budded cells the nucleolar mass started to elongate close to the bud neck, quickly followed by nucleolar segregation along the mother-bud axis (Fig. 6a). In the *int1*ΔΔ *sep7*ΔΔ mutant, we found that most of the binucleated large-budded cells segregated one of the nucleoli to the bud before the synchronous onset of the duplication of both nucleoli, one in each cellular compartment (Fig. 6a). Interestingly, the time-lapse analysis allowed the generation of polynucleated cells in the double mutant to be observed. In some cases, multinucleated cells arose due to defects in cytokinesis that resulted in the formation of trimeras, similar to those described after treatment with fluconazole (FLC)^31^. Cytokinesis failure between a mother-daughter pair was followed by a new budding that generated three cells (mother-daughter-granddaughter) sharing a common cytoplasm with two nuclei. Binucleated cells arose by segregation of four nuclei within three cells compartments (Fig. 6b, cell 1–3). Less frequently, after a normal mitosis in which the two nucleoli segregated to the mother and daughter cells, a second mitosis took place without a new budding, generating two binucleated cells (Fig. 6b, cell 2). Therefore, polyploid cells arose by at least two different mechanisms in *int1*ΔΔ *sep7*ΔΔ cells.

Septins act as a scaffold to recruit factors to the bud neck. In *S. cerevisiae*, Rts1, the B′ regulatory subunit of the PP2A phosphatase, localizes to the cleavage site during cytokinesis and is required for maintaining cellular ploidy^32,33^. Therefore, it could be possible that the polyploid cells observed in the *int1*ΔΔ *sep7*ΔΔ mutant were due to the loss of Rts1 at the bud neck. To investigate this, Rts1-GFP localization was analysed in *int1*ΔΔ, *sep7*ΔΔ and *int1*ΔΔ *sep7*ΔΔ mutants. Quantification of intensity showed no significant differences between the three strains and the wild type (Fig. 6c), indicating that neither Sep7 nor Int1 are required for Rts1 localization at the bud neck. Therefore, the atypical nucleolar segregation of *int1*ΔΔ *sep7*ΔΔ cells was not due to loss of Rts1 from the bud neck.

To confirm whether cytokinesis occurred normally in the *int1*ΔΔ *sep7*ΔΔ mutant, the plasma membrane H(+)-ATPase Pma1 was used. Pma1 decorates the membrane of the cells^34^, allowing the visualization of the double membrane formed between the mother and daughter cells after cytokinesis in the wild type (Fig. 6d). Similar results were observed in the *int1*ΔΔ *PMA1-mCherry* and *sep7*ΔΔ *PMA1-mCherry* strains (not shown). By contrast, in the *int1*ΔΔ *sep7*ΔΔ mutant, trimeras composed by a mother, daughter and granddaughter cells sharing a common cytoplasm could be observed (Fig. 6d). These results indicate a defect in cytokinesis in *int1*ΔΔ *sep7*ΔΔ cells.

**Figure 7:**
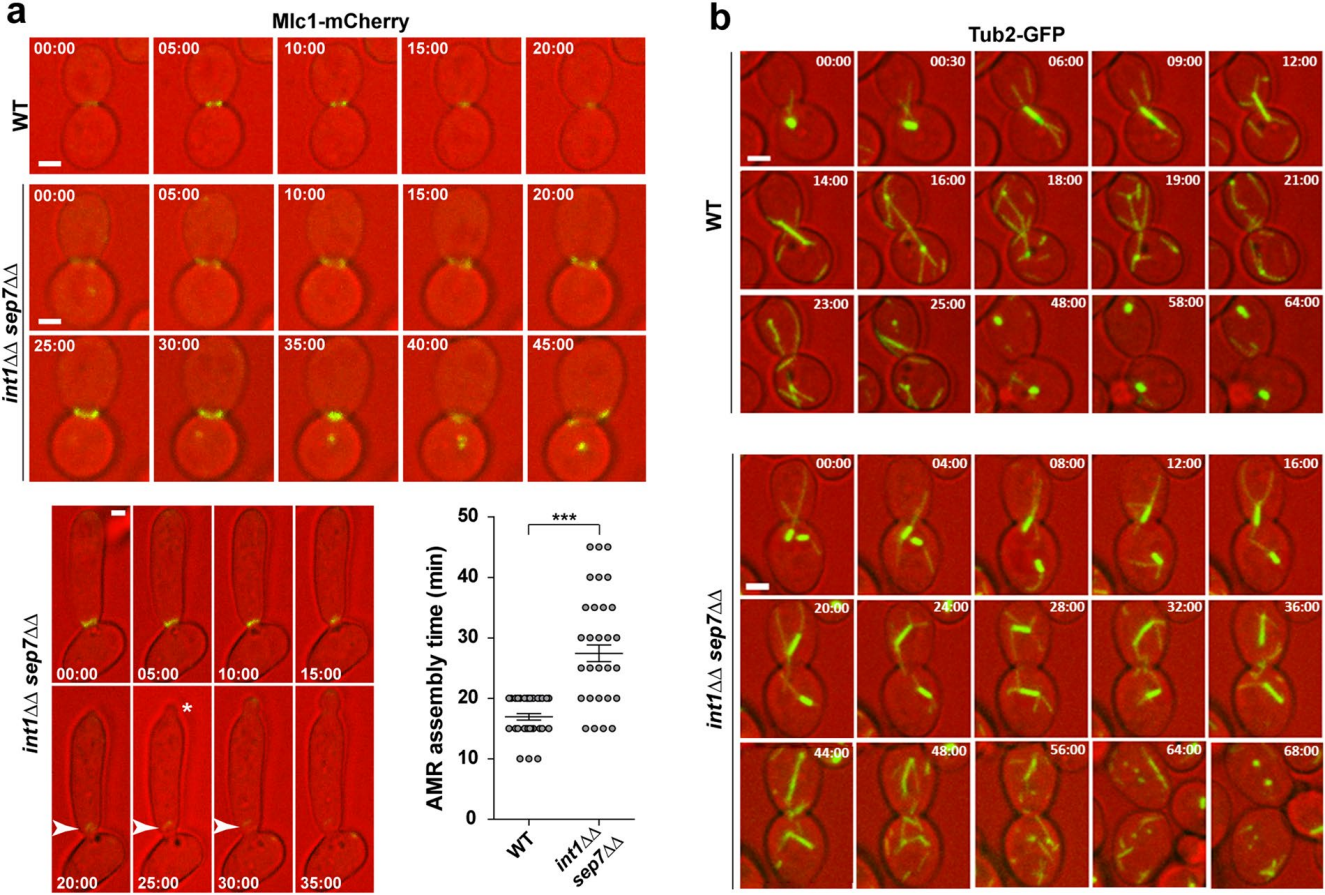
*int1*ΔΔ *sep7*ΔΔ cells have defects in nuclear segregation. (**a**) Time-lapse analysis of Mlc1-Cherry localization in the wild-type (WT, OL2338) and *int1*ΔΔ *sep7*ΔΔ *MLC1-mCherry* (OL2833) strains. Overlay images of DIC (red) and GFP-fluorescence (green) are shown. The images are the maximum projection of 3 z-planes. The numbers indicate time (min:s). Scale bar: 2 μm. The lower panel shows an example of a cell with a defect in AMR contraction in the *int1*ΔΔ *sep7*ΔΔ *MLC1-mCherry* strain at the indicated time points (minutes). Arrowheads indicate the abnormal AMR contraction and the asterisk indicates the apparition of a new bud. The graph shows a quantification of the time of AMR assembly at the bud neck in the same strains, and represents the average time of two independent experiments ± s.e.m (n > 35; ***p-value < 0.0001). (**b**) Time-lapse analysis of Tub2-GFP localization in the wild-type (WT, JC860) and *int1*ΔΔ *sep7*ΔΔ *TUB2-GFP* (OL2536) strains. Overlay images of DIC (red) and GFP-fluorescence (green) are shown. The images are the maximum projection of 3 z-planes. The numbers indicate time (min:s). Scale bar: 2 μm.

Since the above result suggested a cytokinesis failure in *int1*ΔΔ *sep7*ΔΔ cells, AMR contraction was monitored by tagging Mlc1 with m-Cherry. Time-lapse microscopy showed that absence of Int1 and Sep7 produced a marked delay in the time between AMR assembly and contraction (Fig. 7a). Quantification of the time that the AMR was assembled at the bud neck showed a significant difference between the wild type and the *int1*ΔΔ *sep7*ΔΔ mutant (16.9 ± 0.5 minutes versus 27.4 ± 1.4, respectively; p-value < 0.0001, n > 35; Fig. 7a, graph). Interestingly, time-lapse microscopy allowed the observation of cells that failed to correctly contract the AMR and started to assemble a new bud (Fig. 7a, lower panel). Thus, absence of Int1 and Sep7 results in an increase in the time that the AMR is assembled at the bud neck and increases the defects in cytokinesis.

Next, time-lapse microscopy was used to monitor the spindle cycle in *int1*ΔΔ *sep7*ΔΔ cells expressing Tub2-GFP, a microtubule marker. In wild-type large-budded cells, a single Tub2-GFP dot appeared close to the bud neck (T = 00:00) which rapidly assembled a short spindle, visualized as a bar (T = 00:30)(Fig. 7b). The mitotic spindle elongated at anaphase onset (T = 06:00) and within 6 min the mitotic spindle entered the bud, followed by spindle breakdown at the end of mitosis (T = 16:00). After mitosis, Tub2-GFP localized to the SPB until the assembly of a new spindle in the next mitosis (T = 64:00). In bi-nucleated *int1*ΔΔ *sep7*ΔΔ large-budded cells, two short mitotic spindles were assembled close to the bud neck (Fig. 7b, below, T = 0:00). Then, one spindle reached the neck and migrated to the daughter cell (T = 8:00 to 20:00), whereas the other spindle remained in the mother. Then, spindle elongation and breakdown took place in each cell compartment, usually in a coordinated manner, giving rise to G1 cells with two separated nuclei. Therefore, mitotic exit takes place before the spindle enters the neck in *int1*ΔΔ *sep7*ΔΔ cells, suggesting a defective SPOC.

### Deletion of *LTE1* suppresses the segregation defects of *int1*ΔΔ *sep7*ΔΔ mutants

**Figure 8:**
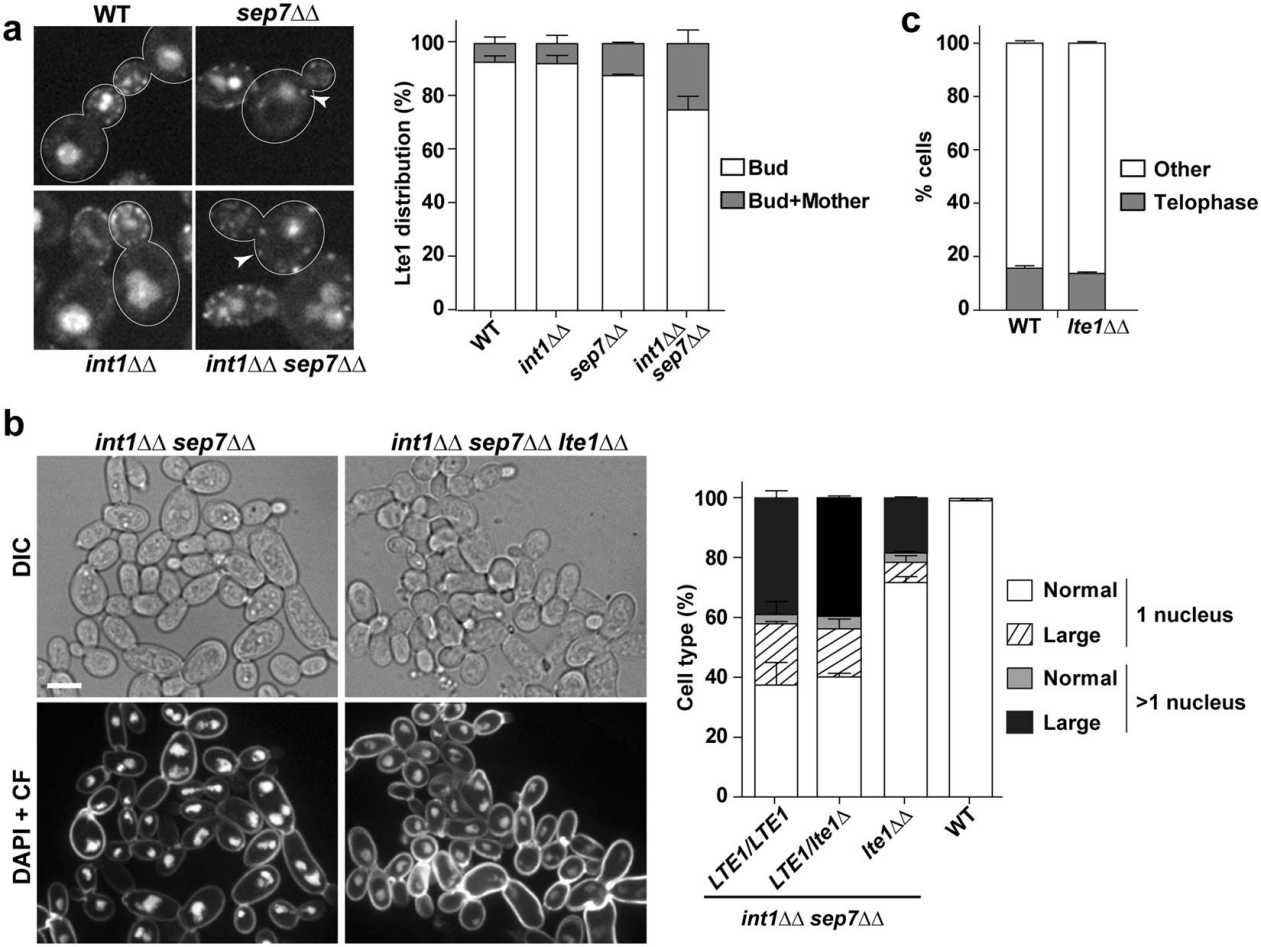
Lte1 is required for abnormal segregation in *int1*ΔΔ *sep7*ΔΔ cells. (**a**) Localization of Lte1-GFP in wild type (WT, OL2689), *sep7*ΔΔ *LTE1-GFP* (OL2691), *int1*ΔΔ *LTE1-GFP* (OL2694) and *int1*ΔΔ *sep7*ΔΔ *LTE1-GFP* (OL2697) cells. A single focal plane is shown. Scale bar: 5 μm. The graph represents the percentage of cells with Lte1 puncta in the bud or in the bud and mother cells and is the average of two independent experiments ± s.e.m. At least 100 small or medium budded cells were counted in each strain. (**b**) Exponentially growing cells of the wild type strain (WT, BWP17) and the *int1*ΔΔ *sep7*ΔΔ (OL2509), *int1*ΔΔ *sep7*ΔΔ *LTE1/lte1*Δ (OL2781) and *int1*ΔΔ *sep7*ΔΔ *lte1*ΔΔ (OL2795) mutants were stained with DAPI and calcofluor (CF). Interferential difference contrast images (DIC) and the maximum projection of 3 z-planes showing DAPI and calcofluor staining are shown. Scale bar, 5 μm. The graph represents the average percentage of cells with one nucleus or more than one nucleus in the same strains from two independent experiments ± s.e.m., and at least 200 cells were counted for each strain. The number of nuclear masses was determined by DAPI staining. (**c**) Deletion of *LTE1* does not delay cell cycle progression. Exponentially growing cells of the *TUB2-GFP* wild-type strain (WT, JC860) and the *lte1*ΔΔ *TUB2-GFP* (OL2818) were imaged and the percentage of cells with telophase spindles was quantified. The graph shows the mean ± s.e.m of two independent experiments in which at least 100 cells were counted for each strain.

In *S. cerevisiae*, Lte1 is an activator of the MEN that localizes to the bud cortex^35,36^. This asymmetric distribution of Lte1 is maintained by the septin ring, which acts as a diffusion barrier between the mother and the bud^11,13^. Thus, it was tested whether the loss of Int1 and Sep7 could disturb the septin-dependent diffusion barrier, allowing Lte1 to be localized into the mother cell to inappropriately activate mitotic exit. To test this hypothesis, *LTE1* was tagged at its C terminus with GFP. In wild-type *C. albicans* cells, Lte1-GFP localized as mobile puncta at the bud cell cortex, although a small percentage of cells (6.9%) showed puncta at the mother cell cortex (Fig. 8a). Loss of Int1 did not significantly modify Lte1 localization. However, deletion of *SEP7* slightly increased Lte1 puncta in mother cells (12%). Interestingly, this percentage was significantly higher in the *int1*ΔΔ *sep7*ΔΔ mutant (24.9%), suggesting that the diffusion barrier defect of *sep7*ΔΔ cells was exacerbated by the loss of Int1.

Because Lte1 acts as a bud-localized MEN activating signal, the diffusion of Lte1-GFP puncta from the bud cortex into the mother in *int1*ΔΔ *sep7*ΔΔ cells could cause inappropriate mitotic exit in this mutant. If this were correct, then deletion of *LTE1* should suppress the inappropriate mitotic exit of *int1*ΔΔ *sep7*ΔΔ cells. To test this idea, we constructed an *int1*ΔΔ *sep7*ΔΔ *lte1*ΔΔ triple mutant and determined the nuclear content by DAPI staining. Loss of one of the *LTE1* alleles in *int1*ΔΔ *sep7*ΔΔ cells did not modify the percentage of polynucleated cells (Fig. 8b). However, deletion of the two *LTE1* alleles largely suppressed the segregation defect of *int1*ΔΔ *sep7*ΔΔ mutant. In the *int1*ΔΔ *sep7*ΔΔ double mutant ~45% of the cells had more than one nucleus per cell, whereas this percentage was reduced to ~21% in the *int1*ΔΔ *sep7*ΔΔ *lte1*ΔΔ triple mutant. Because deletion of *LTE1* did not fully suppress the segregation defect of *int1*ΔΔ *sep7*ΔΔ cells, this observation suggests an Lte1-independent mechanism in the SPOC that is still defective in the double mutant. In *S. cerevisiae*, Lte1 is not essential for cell viability or MEN activation in an unperturbed cell cycle^37^. Mitotic progression of the *lte1*ΔΔ mutant in *C. albicans* was tested by tagging the strain with Tub2-GFP. Similar to what has been described in *S. cerevisiae*, the absence of Lte1 did not produce a telophase arrest at 28 °C (Fig. 8c), suggesting that suppression of the *int1*ΔΔ *sep7*ΔΔ phenotype is not due to a delay in exit from mitosis. Altogether, these results are consistent with Int1 and Sep7 playing a role in the maintenance of cellular ploidy by acting as a diffusion barrier for Lte1.

## Discussion

In animal cells, anillin is a highly conserved multi-domain protein that interacts with several cytoskeleton proteins to ensure that cytokinesis is coordinated with the end of mitosis^16^. In humans and *Drosophila*, anillins interact with septins to promote furrow ingression, septin recruitment and stabilization of the ring^38–40^. Similarly, anillin-like proteins such *S. pombe* Mid2 or *S. cerevisiae* Bud4 are required to maintain the stability of the septin rings during AMR contraction^19,21,41–43^. In *C. albicans*, the anillin-protein Int1 contributes to morphogenesis, adhesion and virulence^25^, and colocalizes with septins in yeast and hyphal cells^26^. Here, we have identified Int1 as a new septin regulator. We show that Int1 localized to the division plane just after septins. Moreover, the presence of septins at the bud neck was required for proper recruitment of Int1 to the division plane. In addition, the PH domain located at the C-terminus of Int1 (aa 1134–1711), which may act as a plasma membrane binding or as a septin interaction domain^20,22^, was also required for localization and function. These observations suggest a functional relationship between Int1 and septins.

Septins have a dual role in cytokinesis. Prior to the onset of cytokinesis, they act as a scaffold to assemble the cleavage apparatus at the bud neck^44^. In late cytokinesis, the double septin ring forms a diffusion barrier to concentrate diffusible cytokinesis factors to the division site^9^. Our results suggest that the cooperation of Int1 with septins in early cytokinesis is not essential, since AMR formation in *int1*ΔΔ cells was apparently normal. However, in late cytokinesis, Int1 was necessary to stabilize the septin ring after duplication both in yeast and hyphae. Time-lapse microscopy of *int1*ΔΔ cells showed a rapid disassembly of septin rings after splitting, with similar kinetics to that of *bud4*Δ mutants in *S. cerevisiae*^19,21,43^. Interestingly, despite the almost complete absence of duplicated septin rings, only a modest delay in cytokinesis was observed in *int1*ΔΔ cells, suggesting that the double septin ring is dispensable for cytokinesis in *C. albicans*. Consistent with our results, cells lacking the double septin ring are able to perform cytokinesis in *S. cerevisiae*^21^. It has been suggested that the dispensability of the septin-dependent diffusion barrier is due to the redundancy between the AMR and the septin double ring in localizing cytokinesis factors to the division plane^21^. Therefore, the absence of cytokinesis defects in cells lacking Int1 might be well explained if this redundant mechanism is also conserved in *C. albicans*.

What may be the role(s) of Int1 in septin regulation in *C*. *albicans*? In *S. cerevisiae*, septins are found in an hourglass structure at the cleavage site during most of the cell cycle until the onset of cytokinesis, when this structure rearranges and splits into two rings distributed on both sides of the AMR. The intensity of Cdc10-GFP in single rings in *int1*ΔΔ cells was similar to that of wild-type cells, suggesting that septins can localize to the bud neck in the absence of Int1. However, this mutant showed a rapid loss of Cdc10 signal concomitant with septin ring duplication before AMR contraction. Since the time-lapse data revealed that the Int1 ring duplicated slightly before the septin ring, this might suggest that Int1 may work as a scaffold protein to stabilize septin filaments during their reorganization from the hourglass structure to duplicated rings. During this process, some of the septin filaments disassemble and reassemble, accompanied by a 90° rotation in orientation^43,45,46^. In addition, as a consequence of AMR contraction, the local membrane curvature changes at the bud neck. Recently, it has been shown that septins have an intrinsic preference to bind to convex membranes *in vitro*^47,48^. Therefore, it could be possible that the interaction between septin filaments and the plasma membrane has to be reinforced at the cytokinesis site during AMR contraction. This function, as a factor necessary to increase the affinity of septins for the membrane, is consistent with the fact that Int1 overexpression in *S. cerevisiae* stabilizes ectopic spiral-like septin structures that co-localize with Int1 in the cell cortex^26^. Furthermore, the expression of the C-terminus of Mid2 in *S. pombe* also stabilizes septin complexes at the plasma membrane^42^. Therefore, the anillin-related proteins have conserved this function in late cytokinesis in many fungi.

In *S. cerevisiae*, a septin-dependent diffusion barrier compartmentalizes the plasma membrane and the cytoplasmic leaflet of the cortical endoplasmic reticulum into distinct domains^9,49^, which is important to generate cell polarity. Among other functions, this compartmentalization provides spatial cues to align the mitotic spindle on the mother-bud axis to guarantee faithful chromosome segregation during mitosis. A cell signalling system, known as SPindle Orientation Checkpoint (SPOC), ensures that cells do not exit mitosis until the mitotic spindle moves into the bud neck^11,14,46^. When this checkpoint is defective, mitotic exit takes place before the spindle enters into the daughter cell, giving rise to a binucleated mother cell and an anucleated bud. A relevant observation of this study is that the *int1*ΔΔ *sep7*ΔΔ mutant showed defects in spindle positioning, producing large cells with more than one nucleus. Interestingly, this defect was absent in the *int1*ΔΔ *cdc10*ΔΔ mutant, suggesting that Int1 collaborates with Sep7 in the SPOC independently of other septin subunits. To our knowledge, this is the first report of a fungal anillin-related protein involved in this checkpoint. However, the mechanism of how Int1 and Sep7 operate in this checkpoint is not clear.

In *S. cerevisiae*, mitotic exit is triggered by the activation of the small G protein Tem1. Tem1 and its GAP Bub2 localize to the daughter SPB whereas the GEF Lte1 is restricted to the bud cortex by a septin-dependent diffusion barrier at the bud neck^13^. This segregation helps to keep mitotic exit inhibited until the spindle enters the bud, where Lte1 is present. Whether the diffusion barriers are required to confine Lte1 to the bud is unclear^50^. In *C. albicans*, we propose that Int1 and Sep7 are required for the formation of a neck diffusion barrier that restricts Lte1 to the daughter cell. We have found that Lte1-GFP localized as cortical puncta at the bud that were highly dynamic. This asymmetric distribution was dependent on Int1 and Sep7, since deletion of these two proteins resulted in an increase in the percentage of cells with Lte1 puncta at the mother cortex when compared to the single mutants or the wild-type strain. Interestingly, deletion of *LTE1* partially rescued the polynucleated phenotype of the *int1*ΔΔ *sep7*ΔΔ mutant. Because deletion of *LTE1* did not affect the timing of mitosis, measured as percentage of mitotic spindles in asynchronous cultures of *LTE1 TUB2-GFP* and *lte1*ΔΔ *TUB2-GFP* strains (Fig. 8c), this partial suppression suggests the existence of two different Int1-Sep7-dependent mechanisms to ensure that each cell receives one nucleus after mitosis. Int1 could collaborate with Sep7 in the formation of an Lte1 diffusion barrier between the mother and bud. In addition, an Lte1-independent mechanism might exist, as 21% of cells displayed more than one nucleus per cell in the *int1*ΔΔ *sep7*ΔΔ *lte1*ΔΔ mutant compared to the 44% in the *int1*ΔΔ *sep7*ΔΔ strain. Since the interaction of astral microtubules (MTs) with the bud neck is required for spindle positioning in *S. cerevisiae*^12^, we speculate that Int1 and Sep7 might interact with an unknown factor, such as Kar9^51,52^, to link astral MTs with septins. Further work will be required to test this idea.

*C. albicans* is an opportunistic human pathogen with a high degree of ploidy plasticity. It is common that clinical isolates of this pathogen are cells with chromosomal aneuploidies and/or polyploidy, and this is frequently associated with drug resistance, increased virulence and pathogenicity in animal models^53,54^. This propensity to develop changes in the karyotype could be an adaptive mechanism to generate variation within the host for overcoming harsh environmental conditions^55^. The progeny of the *int1*ΔΔ *sep7*ΔΔ mutant contained a mixture of normal and large polynucleated cells, but the strain was viable. Interestingly, multinucleated cells arose due to cytokinesis defects leading to the formation of trimeras similar to those described after treatment with FLC^31^, suggesting that FLC might interfere with the same cellular process regulated by Int1 and Sep7. It is known that FLC specifically targets Erg11 and interferes with ergosterol synthesis and membrane integrity^56^. Thus, it is possible that FLC indirectly affects the integrity of the septin ring at the bud neck or its interaction with membrane lipids, resulting in a phenotype similar to that observed in the *int1*ΔΔ *sep7*ΔΔ mutant. Although nuclear segregation and the mechanisms required to maintain cellular ploidy are poorly understood in *C. albicans*, our results suggest that cortical cytoskeletal proteins located at the bud neck are essential for preserving genome stability in this organism.

## Methods

### Strains and growth conditions

The strains used in this work are listed in Supplementary Table 1. Cells were grown in YPD or in synthetic minimal (SC) medium containing the appropriated supplements at 28 °C. Hypha formation was induced by supplementing the media with 10% Fetal Calf Serum (FCS) at 37 °C. Transformation of different strains was performed by electroporation^57^. All transformants were checked for correct genome integration by PCR. Construction of strains carrying disruption of different genes was done using the PCR-mediated procedure previously described and different selectable markers^58^. C-terminal fusion of different fluorescent proteins was performed using pFA plasmids containing GFP or mCherry with different selectable markers as previously described^58,59^. Correct in-frame insertion of the fluorescent proteins was checked by sequencing or microscopic inspection of the transformants.

To construct double and triple deletion mutants, the Ura-blaster technique was used^60^. The coding sequences were replaced by a *hisG-CaURA3-hisG* cassette with flanking regions corresponding to the gene of interest obtained by PCR amplification. Insertion of the cassette was checked by PCR with specific oligonucleotides. Then, Ura^-^ segregants were selected by growing the transformants in plates containing 5-fluoroorotic acid (5-FOA).

### Calculation of cell volume

Yeast cell volume was calculated from the formula for a prolate spheroid 4(πlw^2^)/3, where l is yeast cell length and W is the cell width. We considered large cells those with a volume >1.5 times the average volume of wild-type cells. For each sample, the volumes were determined by counting at least 50 cells from two independent experiments.

### Plasmid constructions

To construct the *int1-*Δ*N* allele, an upstream 5′-region of *INT1* was amplified from genomic DNA and cloned as a *Pvu*II-*Bgl*II fragment into the pFA-CaARG4-MET3 plasmid. Next, the *INT1* promoter region was amplified and used to replace the *MET3* promoter using *Pme*I-*Spe*I sites. Finally, the C-terminal *INT1* coding region (G1127-Q1711) was amplified and cloned into the former plasmid as a *Sal*I-*Spe*I fragment (the *Sal*I site was a synthetically introduced near the *Spe*I site), generating plasmid pC1165. To insert the *int1-*Δ*N* allele into the genome, the whole construct was amplified from pC1165 as a cassette using specific oligonucleotides and used to transform the *INT1*/*int1*Δ strain. To generate *int1-ΔN-GFP* strains, the *INT1/int1-ΔN* strain was transformed with a cassette obtained from pFA-GFP-HIS using oligonucleotides that annealed at the 3′-end of the gene.

### Flow cytometry

For DNA content analysis, cells were prepared as previously described^61^. Basically, cells were fixed with methanol, stained with SybrGreen (1:10000) and analysed on a MACSQuant VYB flow cytometer (Miltenyi Biotech).

### Microscopy

Fluorescence microscopy was performed with a Personal Deltavision microscope running softWoRx (Applied Precision Instruments) equipped with a Photometrics CoolSNAP HQ camera. Z-stack images were collected with step sizes of 0.4 µm and were deconvolved using softWoRx. Images are maximum projections of deconvolved z-stacks. For time-lapse analysis, cells were fixed at the bottom of iBIDI u-slides coated with lectin and covered with the appropriate medium. Samples were observed on an Olympus IX81 microscope equipped with a spinning-disc confocal system (Roper Scientific). Three to five z-planes were collected with step sizes of 0.4 µm. Average fluorescence intensity was quantified with ImageJ (http://rsb.info.nih.gov/ij/) or Metamorph (MDS Analytical Technologies). Septum formation was determined by staining the cells with Calcofluor White at 1 µg/ml final concentration. DNA was stained using 4,6-Diamidino-2-phenylindole (DAPI) at 1 µg/ml final concentration after short treatment of the cells with 1% Triton.

## Electronic supplementary material

Supplementary Information
