# Supplementary material for: The anillin-related Int1 protein and the Sep7 septin collaborate to maintain cellular ploidy in Candida albicans
Source: Sci Rep. 2018 Feb 2;8:2257. doi: 10.1038/s41598-018-20249-9 (PMC5797091; doi:10.1038/s41598-018-20249-9)
Supplement: Supplementary file 1 — Supplementary Information [file 41598_2018_20249_MOESM1_ESM.pdf]

The anillin-related Int1 protein and the Sep7 septin collaborate to maintain cellular ploidy in *Candida albicans*

Sara Orellana-Muñoz[1], Encarnación Dueñas Santero[1],Yolanda Arnáiz-Pita[1], Francisco del Rey[1], Jaime Correa-Bordes[2], Carlos R. Vázquez de Aldana[1,*],

[1] Instituto de Biología Funcional y Genómica, IBFG-CSIC. Universidad de Salamanca. Salamanca, Spain
[2] Departamento de Ciencias Biomédicas. Universidad de Extremadura. Badajoz, Spain
[*] Corresponding author: cvazquez@usal.es

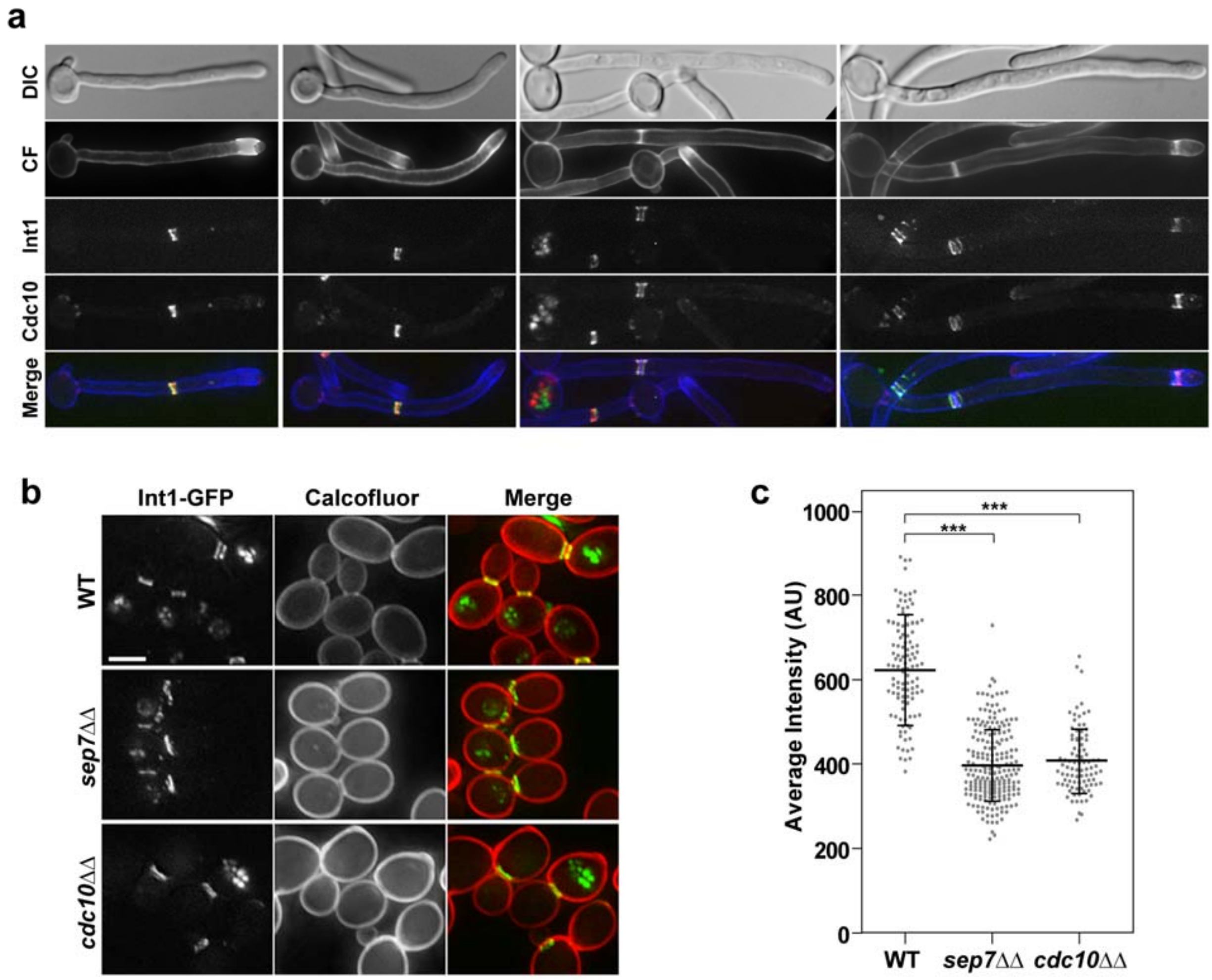

Supplementary Figure S1. Localization of Int1-GFP (a). Localization of Int1-GFP and Cdc10-mCherry during hyphal growth. *INT1-GFP CDC10-mCherry* cells were induced to filament and stained with calcofluor (OL2262). The images are the maximum projection of 10 planes and show the Int1-GFP and Cdc10-mCherry channels and the merged image (Int1-GFP, green; Cdc10-mCherry, red; calcofluor, blue). Scale bar, 2 µm. (b). Images of Int1-GFP and calcofluor staining of the wild-type *INT1-GFP* (OL1611), *sep7*ΔΔ *INT1-GFP CDC10-mCherry* (OL2310) and *cdc10*ΔΔ *INT1-GFP* (OL1644) strains. The merged channel shows Int1-GFP (green) and calcofluor (red). The images are the maximum projection of 10 planes. Scale bar, 2 µm. (c). Int1 localizes more poorly in *sep7*ΔΔ and *cdc10*ΔΔ mutants. Average intensity of Int1-GFP rings in the wild type *INT1-GFP*, *sep7*ΔΔ *INT1-GFP CDC10-mCherry* and *cdc10*ΔΔ *INT1-GFP* strains, with the standard error. Two independent experiments were performed (n>90 rings). ***. p-value < 0.0001.

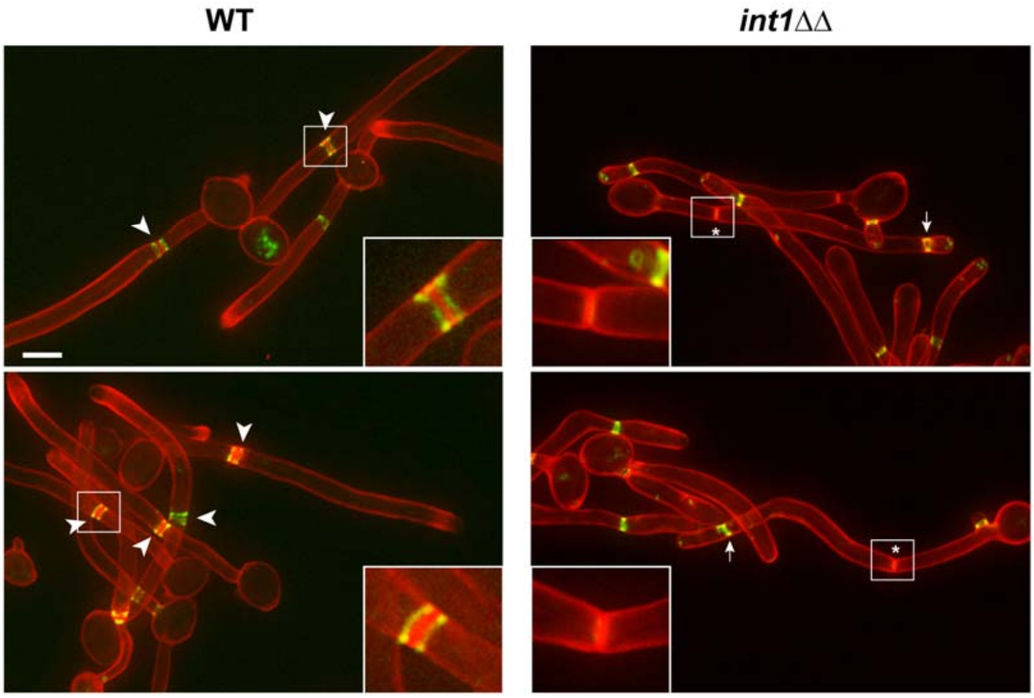

Supplementary Figure S2. Int1 is also required for septin ring stability during hyphal growth. Images of *CDC10-GFP* (OL2243) and *int1*ΔΔ *CDC10-GFP* (OL2316) hyphae after 2.5 h of induction. The images are the maximum projection of 10 z-planes and show the merged signal of Cdc10-GFP (green) and calcofluor staining (red). Scale bar, 5 µm. Arrows indicate visible apical septin rings, arrowheads point to old septa with septin rings and asterisks mark septa without visible Cdc10-GFP signal. The regions marked with a rectangle have been magnified.

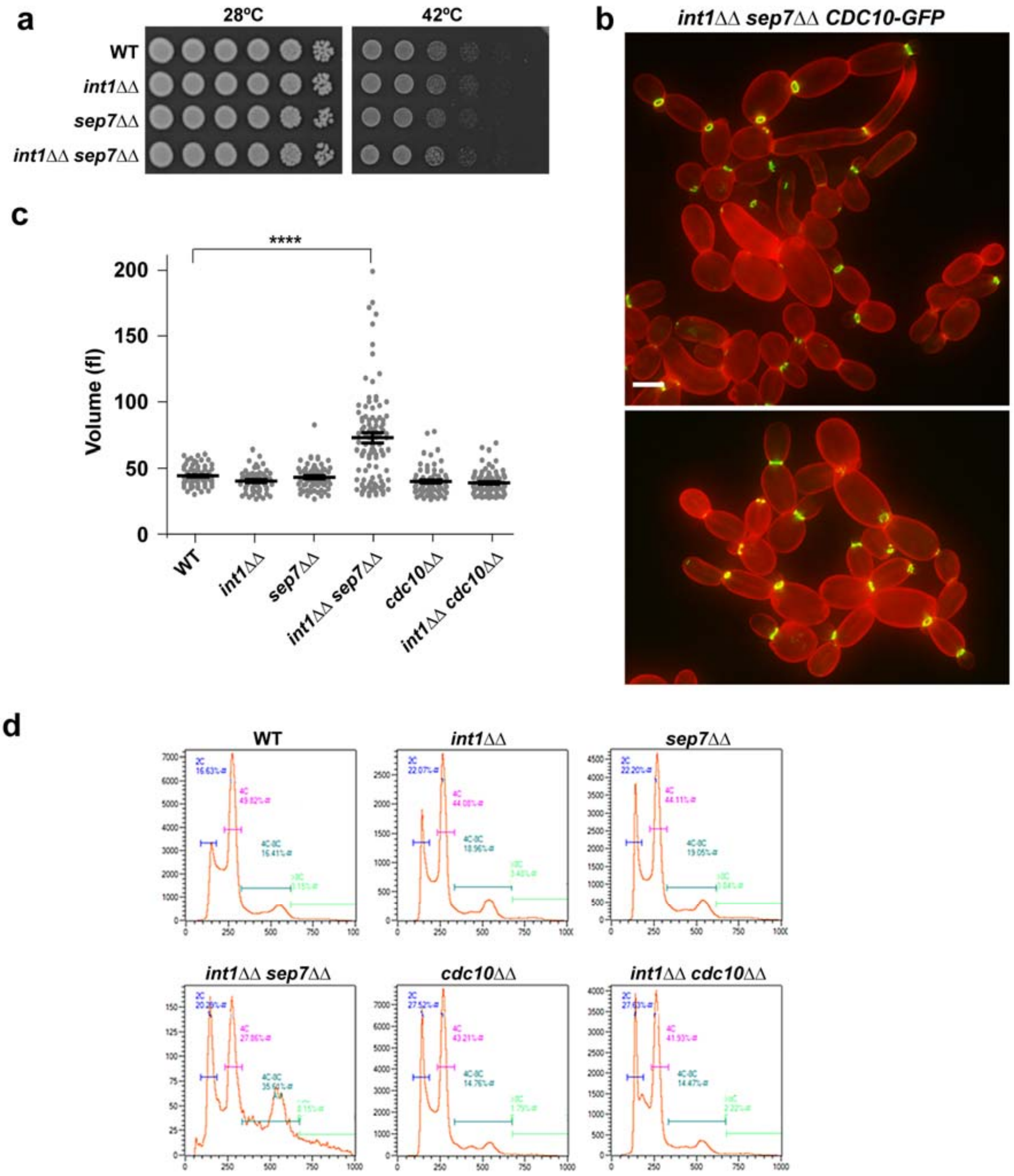

Figure S3. Combinations of deletion in *INT1*, *SEP7* and *RTS1* generate cells with high DNA content. (a). Growth of several single and double mutants at different temperatures. Serial dilutions were plated on YEPD medium and incubated at the indicated temperatures for 2 days. Same strains as in Figure 5. **(b)** Localization of Cdc10-GFP in the *int1*ΔΔ *sep7*ΔΔ *CDC10-GFP* (OL2513) mutant during yeast growth. The images are the maximum projection of 10 planes acquired every 0.4 µm and show the merged signal of Cdc10-GFP (green) and calcofluor staining (red). Scale bar, 5 µm. **(c)** Cell volumes of indicated strains grown exponentially in YPD at 28ºC. The results are pooled data from two independent experiments (n>50/experiment). ****, p-value<0.0001. **(d)** Flow cytometry analysis of the wild-type BWP17 (WT), *int1*ΔΔ (OL2314), *sep7*ΔΔ (OL2138), *int1*ΔΔ *sep7*ΔΔ (OL2509), *cdc10*ΔΔ (OL2213) and *int1*ΔΔ *cdc10*ΔΔ (OL2532) strains.

Supplementary Table S1. Strains used in this study

Strain	Name	Genotype	Origin
BWP17	WT	*uraΔ::imm434/ura3Δ::imm434, his1Δ::hisG/his1Δ::hisG, arg4Δ::hisG/arg4Δ::hisG*	Enloe *et al.*, 2000
JC860	*TUB2-GFP*	*TUB2-GFP::URA3*	J. Correa
OL1611	*INT1-GFP*	*INT1/INT1-GFP::HIS1*	This study
OL1644	*cdc10ΔΔ INT1-GFP*	*cdc10Δ::hisG/cdc10Δ::hisG INT1-GFP::URA3*	This study
OL2138	*sep7ΔΔ*	*sep7Δ::SAT1/sep7Δ::HIS1*	This study
OL2193	*int1-ΔC CDC10-GFP*	*int1-ΔC (1135-1712)-HA::URA3/int1Δ::SAT1 CDC10/CDC10-GFP::HIS1*	This study
OL2213	*cdc10ΔΔ*	*cdc10Δ::SAT1/cdc10Δ::HIS1*	This study
OL2243	*CDC10-GFP*	*CDC10/CDC10-GFP::ARG4*	This study
OL2262	*INT1-GFP CDC10-Ch*	*INT1/INT1-GFP::HIS1 CDC10/CDC10-mCherry::URA3*	This study
OL2278	*int1-ΔC-GFP CDC10-Ch*	*int1-ΔC (1134-1712)-GFP::HIS1/int1Δ::SAT1 CDC10/CDC10-mCherry::URA3*	This study
OL2280	*int1-ΔN-GFP CDC10-Ch*	*ARG4::int1-ΔN (1-1126)-GFP::HIS1/int1Δ::SAT1 CDC10/CDC10-mCherry::URA3*	This study
OL2304	*int1-ΔN CDC10-GFP*	*ARG4::int1-ΔN (1-1126)-HA::URA3/int1Δ::SAT1 CDC10/CDC10-GFP::HIS1*	This study
OL2310	*sep7ΔΔ INT1-GFP CDC10-Ch*	*sep7Δ::SAT1/sep7Δ::ARG4 INT1/INT1-GFP::HIS1 CDC10/CDC10-mCherry::URA3*	This study
OL2314	*int1ΔΔ*	*int1Δ::SAT1/int1Δ::ARG4*	This study
OL2316	*int1ΔΔ CDC10-GFP*	*int1Δ::SAT1/int1Δ::ARG4 CDC10/CDC10-GFP::URA3*	This study
OL2328	*int1ΔΔ CDC10-GFP MLC1-Ch*	*int1Δ::SAT1/int1Δ::ARG4 CDC10/CDC10-GFP::URA3 MLC1/MLC1-mCherry::HIS1*	This study
OL2338	*CDC10-GFP MLC1-Ch*	*CDC10/ CDC10-GFP::ARG4 MLC1/MLC1-mCherry::HIS1*	This study
OL2347	*NOP1-Ch*	*NOP1/NOP1-mCherry::URA3*	This study
OL2509	*int1ΔΔ sep7ΔΔ*	*int1Δ::SAT1/int1Δ::ARG4 sep7Δ::hisG/sep7Δ::URA3*	This study
OL2513	*int1ΔΔ sep7ΔΔ CDC10-GFP*	*int1Δ::SAT1/int1Δ::ARG4 sep7Δ::hisG/sep7Δ::URA3 CDC10/CDC10-GFP::HIS1*	This study
OL2514	*int1ΔΔ sep7ΔΔ NOP1-Ch*	*int1Δ::SAT1/int1Δ::ARG4 sep7Δ::hisG/sep7Δ::URA3 NOP1/NOP1-mCherry::HIS1*	This study
OL2532	*int1ΔΔ cdc10ΔΔ*	*int1Δ::SAT1/int1Δ::ARG cdc10Δ::HIS1/cdc10Δ::URA3*	This study
OL2536	*int1ΔΔ sep7ΔΔ TUB2-GFP*	*int1Δ::SAT1/int1Δ::ARG4 sep7Δ::hisG/sep7Δ::URA3 TUB2/TUB2-GFP::HIS1*	This study
OL2550	*RTS1-GFP*	*RTS1/RTS1-GFP:: HIS1*	This study
OL2552	*sep7ΔΔ RTS1-GFP*	*sep7Δ::SAT1/sep7Δ::HIS1 RTS1/RTS1-GFP::URA3*	This study
OL2553	*int1ΔΔ RTS1-GFP*	*int1Δ::SAT1/int1Δ::ARG4 RTS1/RTS1-GFP::HIS1*	This study
OL2555	*int1ΔΔ sep7ΔΔ RTS1-GFP*	*int1Δ::SAT1/int1Δ::ARG4 sep7Δ::hisG/sep7Δ::URA3 RTS1/RTS1-GFP::HIS1*	This study
OL2689	*LTE1-GFP*	*LTE1-GFP::URA3*	This study
OL2691	*sep7ΔΔ LTE1-GFP*	*sep7::SAT1/sep7::HIS1 LTE1-GFP::URA3*	This study
OL2694	*int1ΔΔ LTE1-GFP*	*int1::SAT1/int1::ARG4 LTE1-GFP::URA3*	This study
OL2697	*int1ΔΔ sep7ΔΔ LTE1-GFP*	*int1Δ::SAT1/int1Δ::ARG4 sep7Δ::hisG/sep7Δ::URA3 LTE1-GFP::HIS1*	This study
OL2732	*int1ΔΔ CDC12-GFP*	*int1Δ::SAT1/int1Δ::ARG4 CDC12-GFP::URA3*	This study
OL2734	*int1ΔΔ SEP7-GFP*	*int1Δ::SAT1/int1Δ::ARG4 SEP7-GFP::URA3*	This study
OL2755	*PMA1-mCherry*	*PMA1/PMA1-mCherry::HIS1 CBK1/CBK1-YFP::URA3*	This study
OL2781	*int1ΔΔ sep7ΔΔ LTE1/lte1Δ*	*int1Δ::SAT1/int1Δ::ARG4 sep7Δ::hisG/sep7Δ::hisG LTE1/lte1Δ::HIS1*	This study
OL2783	*int1ΔΔ sep7ΔΔ PMA1-mCherry*	*int1Δ::SAT1/int1Δ::ARG4 sep7Δ::hisG/sep7Δ::URA3 PMA1/PMA1-mCherry::HIS1*	This study

OL2795	*int1ΔΔ sep7ΔΔ lte1ΔΔ*	*int1Δ::SAT1/int1Δ::ARG4 sep7Δ::hisG/sep7Δ::hisG lte1Δ::URA3/lte1Δ::HIS1*	This study
OL2818	*lte1ΔΔ TUB2-GFP*	*lte1Δ::HIS1/lte1Δ::URA3, TUB2/TUB2-GFP::ARG4*	This study
OL2833	*int1ΔΔ sep7ΔΔ MLC1-Cherry*	*int1Δ::SAT1/int1Δ::ARG4 sep7Δ::hisG/sep7Δ::URA3 MLC1-mCherry::HIS1*	This study
OL2838	*sep7ΔΔ PMA1-mCherry*	*sep7Δ::hisG/sep7Δ::URA3 PMA1/PMA1-mCherry::HIS1*	This study
OL2840	*int1ΔΔ PMA1-mCherry*	*int1Δ::SAT1/int1Δ::ARG4 PMA1/PMA1-mCherry::HIS1*	This study

Enloe B, Diamond A, Mitchell AP. 2000. A single-transformation gene function test in diploid *Candida albicans*. J Bacteriol 182:5730-6.
